# Rumen Microbiota in Cattle and Buffaloes: Insights into Host-Specific Bacterial Diversity

**DOI:** 10.3390/biology14091166

**Published:** 2025-09-01

**Authors:** Shyam Sundar Paul, Avijit Dey, Daoharu Baro, Jerome Andonissamy, Jyotirmoyee Paul, Balbir Singh Punia

**Affiliations:** 1 Division of Animal Nutrition and Feed Technology, ICAR—Central Institute for Research on Buffaloes, Sirsa Road, Hisar 125001, Haryana, India; 2 Division of Animal Physiology and Reproduction, ICAR—Central Institute for Research on Buffaloes, Sirsa Road, Hisar 125001, Haryana, India; 3 School of Computer Science and Engineering, Vellore Institute of Technology, Vellore 632014, Tamil Nadu, India

**Keywords:** bacteria, rumen, diversity, buffalo, cattle, meta-analysis

## Abstract

The present investigation was designed to elucidate the comparative collective diversity of bacteria in the rumen of buffalo and cattle. For this study, a total of 14,913 16S rRNA gene (rrn) sequences generated through Sanger sequencing of ruminal bacteria deposited in the GenBank database were analyzed, of which 13,432 sequences were from cattle and 1481 sequences were from buffalo. Bacterial sequences of cattle origin represented 18 existing phyla and 165 genera, and those of buffalo origin represented 12 phyla and 67 genera. According to Ribosomal Database Project (RDP) classifier, Firmicutes was the dominant phylum in cattle, representing 47.9% of all sequences. Bacteroidetes was the second most abundant phylum (32.3% of sequences), while Proteobacteria accounted for 8.6% of total sequences. In buffalo, Firmicutes was the dominant phylum with 47.2% of total sequences. Bacteroidetes and Proteobacteria phyla constituted 38.3% and 4.6% of total sequences, respectively. We identified 172 shared non-rare species-level operational taxonomic units (OTUs) between buffalo and cattle, with 17 unique to buffalo belonging to three phyla: Bacteroidetes, Firmicutes, and Fibrobacteres. In cattle, 774 OTUs of unique sequences were assigned to six phyla, namely, Firmicutes (422 OTUs), Bacteroidetes (234 OTUs), Fibrobacteres (99 OTUs), Actinobacteria (7 OTUs), Cyanobacteria (5 OTUs), and SR1 (7 OTUs). This study revealed significant differences in rumen bacterial diversity between buffaloes and cattle, supporting the development of species-specific strategies to enhance fibrous feed utilization.

## Simple Summary

The rumen microbiome of buffaloes and cattle differs in diversity owing to their host-specific feed habits and behavior. A few studies have been undertaken to compare diversity of rumen bacteria of cattle and buffalo using MiSeq methods. Typically, individual studies analyzed the rumen content of four to six animals from each species that fed same diet from one geographical location. Such studies were unlikely to achieve high coverage of bacterial diversity due to their narrow scope with respect to number of sampled animals, feeds, and geographic regions. Many rrn sequences recovered from the rumen using Sanger sequencing were deposited in GenBank by researchers from different geographical regions. Therefore, a global meta-analysis of rrn sequences available in GenBank was performed to obtain better collective insight into comparative diversity and census of ruminal bacteria in cattle and buffalo. The genera *Butyrivibrio*, *Prevotella*, *Psychrobacter*, and *Fibrobacter* were documented as most predominant in cattle; however, *Succiniclasticum*, *Prevotella*, *Rumenobacter*, and *Fibrobacter* were the most predominant in buffalo. This study generated substantial information on the diversity of bacteria in the rumen of buffaloes and cattle and demonstrated large variations between the species, which could enable the development of species-specific strategies for the efficient utilization of fibrous feeds.

## 1. Introduction

The rumen, a specialized stomach chamber in herbivores, hosts a complex microbial ecosystem, where diverse microorganisms interact to digest lignocellulosic feeds. Different feed stuffs and agricultural by-products can significantly alter both the microbial population and ruminal fermentation parameters [1,2]. The composition of microbes is controlled by many factors such as the host, feed, rumen pH, etc. [3,4,5]. The environmental condition of the rumen of livestock is strictly anaerobic and is adapted for maintaining a large and diverse microbial population. Within the rumen microbiome, bacteria occupy a major part of the population at 10^10^ to 10^11^ g^−1^ of the content and make the greatest contribution to the digestion of feeds [6]. The diverse bacterial population in the rumen support the animals in the digestion and utilization of lignocellulosic feeds [7]. Numerous efforts have been made to modulate rumen functions in order to improve fiber digestion or reduce nitrogen excretion, but the success of such efforts remained largely limited primarily due to lack of sufficient understanding of the complexity and diversity of the rumen microbiome. The ruminal microbiome was investigated mainly using culture-based methods until the 1980s [8,9,10]. However, it became evident that about 90% of rumen bacteria were not culturable [11]. Thereafter, many studies attempted to characterize the ruminal microbiome using culture-independent methods like molecular hybridization probe real-time PCR [12,13,14], denaturing gradient gel electrophoresis [15], restriction fragment length polymorphism (RFLP) [16], single-strand conformation polymorphism [17], suppressive subtractive hybridization [18], Sanger sequencing of 16S/18S rRNA gene (rrn) [19] sequences recovered in clone libraries prepared from PCR amplification of metagenomic DNA to obtain insight into the rumen ecosystem.

Subsequently, next-generation sequencing [20,21,22] technologies have been adopted to characterize rumen microbes because of high depth coverage and ease of use. Generally, sequence data of rrns generated through Sanger sequencing has much lower error rate and higher length coverage than the next-generation sequencing data and hence are expected to provide better taxonomic resolution.

Buffaloes (*Bubalus bubalis*) are vital to small holders’ farming systems in Asia, providing milk, meat, and drought power. They have a larger rumen than cattle and a strong microbial system, which enables them to digest fibrous feed materials more efficiently than cattle [23,24]. The feeding behavior of buffaloes also differs in comparison to cattle. It was hypothesized that the rumen bacterial community, which mostly depends on host, feeds, and feeding behavior of a species might differ between cattle and buffaloes [25,26]. A few studies [27,28,29,30] have been undertaken to compare diversity of rumen bacteria of cattle and buffalo using MiSeq sequencing methods. Typically, individual studies analyzed rumen content of four to six animals from each species that were fed one feed from one geographical location. Such studies were unlikely to achieve high coverage of bacterial diversity due to their narrow scope with respect to number of sampled animals, feeds, and geographic regions. Many rrn sequences recovered from the rumen using Sanger sequencing (known to have lower sequencing error rate and higher length than those generated through next-generation sequencing technologies) were deposited in GenBank by researchers from different geographical regions but have not been reported in the literature, contributing little to understanding rumen microbial diversity. We hypothesized that a global meta analysis of rrn sequences generated through Sanger sequencing available in GenBank can provide better collective insight into comparative diversity and a census of ruminal bacteria in cattle and buffalo than that indicated by individual studies. This study, therefore, was planned to examine the global diversity of rumen bacteria of cattle and buffalo to gain comprehensive insight into complexity of the ruminal bacteria in these two species.

## 2. Materials and Methods

### 2.1. Collection of Sequence Datasets

All the rumen-origin bacterial 16S rRNA sequences for both cattle and buffalo were obtained from the GenBank database (http://www.ncbi.nlm.nih.gov/genbank). The search term ‘Bacteria [ORGN] AND (cattle OR cow OR (*Bos indicus*) OR (*Bos taurus*)) AND (rumen OR ruminal) AND (16S rRNA [TITL] OR 16S ribosomal RNA [TITL])’ was used for retrieving bacterial 16S rRNA sequences of cattle rumen. Similarly, the search term ‘Bacteria [ORGN] AND (buffalo OR (*Bubalus bubalis*)) AND (rumen OR Ruminal) AND (16S rRNA [TITL] OR 16S ribosomal RNA [TITL])’ was used to retrieve 16S rRNA sequences of buffalo rumen.

Using MOTHUR software version 1.39.0 [31], we subjected the sequences to a quality check using the screen.seqs function of MOTHUR, where sequences shorter than 250 bp (to filter out potentially less informative shorter sequences) and longer than 1600 bp (to filter out irrelevant sequences) as well as sequences with ambiguous and high homopolymer bases (>8) were removed. We detected and removed chimeric sequences using Uchime within MOTHUR pipeline. The sequences were further aligned with bacterial type species *E. coli* (GenBank: X80725.1) to affirm 16s rRNA origin. The collected sequences were classified taxonomically using RDP classifier (https://sourceforge.net/projects/rdp-classifier/). All the rrn sequences of both cattle and buffaloes (>250 bp) were aligned against the rrn Green gene database [32]. The resulting aligned sequences were inserted into the Green gene database ARB tree to generate a detailed phylogenetic tree using the positional variance by parsimony method [33].

### 2.2. Diversity Estimate

The sequences were aligned with multiple sequence alignment program, MAFFT version 7 [34]. For operational taxonomic unit (OTU) binning, a maximum likelihood phylogenetic tree was constructed with generalized time-reversible (GTR) models of nucleotide evolution using FastTree. OTUs were calculated using TreeChopper algorithm (available at https://microbiomeutil.sourceforge.net/), which utilizes phylogenetic trees to bin sequences into OTUs according to the phylogenetic distance. MOTHUR-compatible list file was prepared from TreeChopper output, and OTUs shared between cattle and buffalo were analyzed using MOTHUR. OTUs were classified using Silva Bacteria reference sequences. Rarefaction was also calculated using the MOTHUR program for the rarefaction curve. To estimate asymptote, an estimate of expected maximum species richness complementary to the ACE (abundance-based coverage estimator) and Chao1 richness estimators, the following monomolecular model was fitted to the rarefaction output using the nonlinear least square method of curve fitting with GraphPad Prism software version 6 (available at www.graphpad.com):

OTUs = α(1 − β · e ^[−k · n]^)

where α (asymptote), β, and k values are all derived from the rarefaction analysis, and n represents the number of sequences [35,36]. Rarefaction and other alpha diversity indices (ACE, Chao1, Shannon, and Simpson indices) were calculated with as well as without rare OTUs (singleton and doubletons) using the MOTHUR program.

## 3. Results

A meta-analysis of the ruminal bacteria of cattle and buffalo was conducted using all publicly available rrn sequences that have been recovered worldwide using Sanger sequencing technology.

### 3.1. Data Summary

In total, 14,913 sequences of bacteria were analyzed, of which 13,432 sequences were of cattle and 1481 sequences of buffalo rumen origin. Bacterial sequences from cattle represented 18 phyla, while those from buffalo represented 12 phyla. These phyla comprised 165 genera in cattle and 67 genera in buffalo. Firmicutes was the major phylum, followed by Bacteroidetes and Probacteria in both species.

### 3.2. Taxonomic Classification of Cattle Sequences

Results of taxonomic classification of all the sequences by RDP classifier are presented in the following section. The RDP classifier classified most of the sequences to the genus level, as its reference training set contained taxonomic information down to the genus level.

#### 3.2.1. Major Phyla

##### Firmicutes

About 47.9% of the total sequences were assigned to the phylum Firmicutes. Firmicutes was represented by 6436 sequences. Phylum Firmicutes comprised four classes, of which class Clostridia was the largest, representing 85.6% of the total sequences, followed by Negativicutes (7.02%), Bacilli (4.2%) and Erysipelotrichia (1.27%). The Firmicutes sequences were assigned to a total of 71 genera. Within the class Clostridia, sequences were assigned to 41 genera, and class Bacilli was represented by 17 genera. The class Negativicutes was represented by nine genera, and the class Erysipelotrichia was represented by four genera.

In class Clostridia, *Butyrivibrio* (4.9% of Firmicutes) was the largest genus, comprising 315 sequences. In class Negativicutes, *Succiniclastticum* was the dominant genus (3.2% of the Firmicutes). In class Bacilli, *Streptococcus* was the dominant genus (1.4% of Firmicutes), while *Sharpea* predominated in Erysipelotricha (0.5% of Firmicutes). In addition to these genera, other important genera included *Mogibacterium* (2.3% of Firmicutes), *Pseudobutyrivibrio* (2.8% of Firmicutes), *Saccharofermentans* (2.4% of Firmicutes), and *Ruminococcus* (2.9% of Firmicutes).

##### Bacteroidetes

The Proteobacteria phylum accounted for 8.6% of total sequences (1151 sequences). This Phylum was represented by five classes. The class Epsilonproteobacteria (0.7%) was assigned to two genera; however, Betaproteobacteria (6.8%), Alphaproteobacteria (4.8%), Deltaproteobacteria (8.51%), and Gammaproteobacteria (76.4%) were assigned to 13, 7, 4, and 22 genera, respectively. Among the genera, *Psychrobacter* (25.9% of Proteobacteria) was the most predominant genus, and 299 sequences were assigned under the family Moraxellaceae of class Gammaproteobacteria. Genus *Desulfovibrio* (2.3% of Proteobacteria), *Vampirovibrio* (4.3% of Proteobacteria) of family Desulfbacterales, Pseudomonas (2.0%) of family Pseudomonadaceae, and genus *Shigella*/*Escherichia* (4.9%) of family Enterobacteriaceae were also abundant.

##### Proteobacteria

Proteobacteria comprised 1151 sequences, contributing 8.6% to the total sequences. This phylum was represented by five classes. Class Epsilonproteobacteria (0.7%) was assigned to two genera; however, 13, 7, 4 and 22 genera were assigned to Betaproteobactria (6.8%), Alphaproteobacteria (4.8%), Deltaproteobacteria (8.51%), and Gammaproteobacteria (76.4%), respectively. Among the genera, *Psychrobacter* (25.9% of phylum) was the predominant genus, with 299 sequences under the family Moraxellaceae of class Gammaproteobacteria. Genus *Desulfovibrio* (2.3% of phylum), Vampirovibrio (4.3%) of family Desulfbacterales, Pseudomonas (2.0%) of family Pseudomonadaceae, and genus *Shigella*/*Escherichia* (4.9%) of family Enterobacteriaceae were also abundant.

#### 3.2.2. Minor Phyla

The phyla accounting for <2% of the total sequences were considered minor phyla. Phyla Synergistetes (1.9%), Verrucomicrobia (1.5%), Fibrobacteres (1.4%), Spirochaetes (1.2%), and Actinobacteria (0.5%) were the most common among the minor phyla. The remaining phylum included SR1 (34 sequences), Armatimonadetes (4), Cyanobacteria (1), Fusobacteria (12), Chloroflexi (9), candidates Saccharibacteria (32), Tenericutes (30), Planctomycetes (27), Elusimicrobia (29), Lentisphaerae (59). A total of 33 genera were assigned to minor phyla. The phylum Actinobacteria accounted for 11 genera. Other genera under the minor phylum included *Pyramidobacter* (46 seq) of phylum *Synergistetes*, *Fibrobacter* (186 seq) of phylum Fibrobacteres, and *Victivallis* (50 seq) of phylum Lentisphaerae.

### 3.3. Taxonomic Classification of Buffalo Sequences

#### 3.3.1. Major Phyla

##### Firmicutes

Firmicutes was the most predominant phylum accounting for 47.4% of the total sequences. The phylum comprised three classes: Bacilli (8.1% of Firmicutes sequences), Negativicutes (11.8%), and Clostridia (77.2%). The class Clostridia contained 23 genera, in which the genus *Ruminococcus* was the largest (3.6% of phylum). Negativicutes was the second major class, represented by six genera. *Succiniclasticum* (4.7% of phylum) was the major genus under the phylum Negativicutes. The class Bacilli was assigned six genera, and the genus Staphylococcus (4.1% of phylum) was the most predominant genus within the class Bacilli. In addition to these, genus *Selenomonas* (2.4% of phylum) of class Negativicutes and Clostridium (3.3%), Butyrivibrio (3.4%), Pseudobutyrivibrio (3.7%), Saccharofermentans (3.3% of phylum) of class Clostridia were also assigned large numbers of sequences.

##### Bacteroidetes

Bacteroidetes constituted 568 sequences that were assigned with single major class, Bacteroidia (89.9%), and the remaining sequences were unclassified at the class level. Nine genera were assigned to the class, and the genus *Prevotella* (31.7% of phylum) was the main one among all genera. Other remaining genera included *Bacteroides* (0.4% of phylum), *Rikenella* (1.9%), *Parabacteroides* (1%), *Dysgonomonas* (0.9%), *Paraprevotella* (0.7%), and *Hallella* (0.9%).

##### Proteobacteria

This phylum constituted 4.6% of the total sequences and was classified into five classes: Epsilonproteobacteria (4.4% of phylum Proteobacteria sequences), Betaproteobacteria (8.2%), Deltaproteobacteria (16.7%), Alphaproteobacteria (13.2%), and Gamma proteobacteria (51.5%). The phylum was assigned 13 genera, of which genus *Vampirovibrio* (11.8% of phylum) under class Deltaproteobacteria, *Ruminobacter* (13.2% of phylum) and *Escherichia*/*Shigella* (10.3%) under Gamma proteobacteria were the major genera.

#### 3.3.2. Minor Phyla

These included the phyla in which the sequence coverage was below 4%. These included a total of nine phyla, which represented 10 genera. Phyla Fibrobacteres (3.5%), Verrucomicrobia (0.8%), Lentisphaerae (0.5%), and Spirochaetes (0.5%) were predominant among the minor phyla. The remaining phyla included Elusimicrobia (two), Fusobacteria (two), Candidatus Saccharibacteria (two), Tenericutes (one), and Planctomycetes (one). Genus *Fibrobacter* (52 sequences) of phylum Fibrobacteres was the most highly represented genus.

### 3.4. Diversity Estimates

Results of estimation of different alpha diversity metrices are presented in Table 1. The species richness indices such as abundance-based coverage estimator (ACE) and Chao1 were much lower in buffalo than in cattle. The diversity estimators that take into account both richness and evenness such as Simpson and Shannon were high in both species.

**Figure 1:**
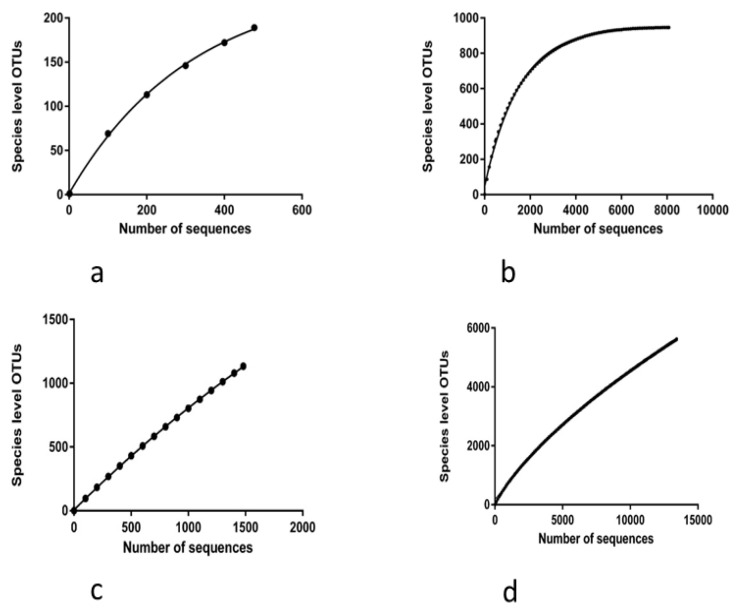
Rarefaction curves for bacterial sequences: (**a**) non-singleton, non-doubleton OTUs from rumen of buffalo; (**b**) non-singleton, non-doubleton OTUs from rumen of cattle; (**c**) all OTUs from rumen of buffalo; (**d**) all OTUs from rumen of cattle.

The rarefaction curve depicts the correlation between the number of sequences and the number of OTUs (Figure 1), where the steeper the slope, the higher the diversity. The rarefaction curve for non-rare OTUs approached the asymptotic level in case of cattle (Figure 1b). Asymptote estimation from rarefaction analysis of non-rare cattle sequences showed 99.6% sequence coverage, and buffalo sequences showed 78.9% coverage at the species level (Table 1).

The Chao and ACE estimates were higher than the rarefaction estimate (Table 1).

**Figure 2:**
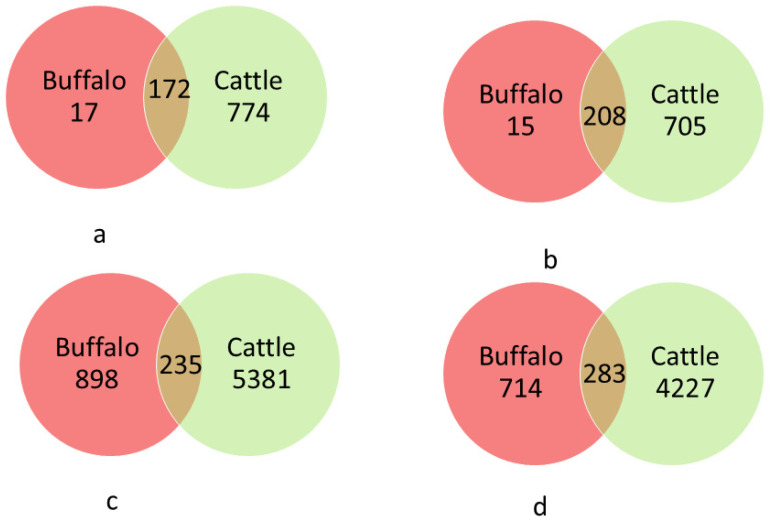
Venn diagram showing unique and shared OTUs: (**a**) non-rare species-level OTUs (each having at least 3 members) at 0.03 16S rRNA gene distance; (**b**) non-rare-genus level OTUs at 0.05 16S rRNA gene distance level; (**c**) all OTUs at species level; (**d**) all OTUs at genus level.

Venn diagrams depicting the extent of overlap of OTUs between the two hosts are presented in Figure 2. The Venn diagram indicated a total microbial richness of 963 OTUs. The number of species-level OTUs in the group buffalo was 189, and in cattle it was 946.

The number of non-rare species-level OTUs shared between groups of buffalo and cattle was 172 (Figure 2a). The percentage of species-level OTUs shared among groups buffalo and cattle was 17.9%. Taxonomic analysis of representative sequences of OTUs using RDP classifier indicated that the shared OTUs were grouped into five phyla. Firmicutes were the largest phylum, assigned 95 OTUs, and consisted of two classes Bacilli (5 OTUs), and Clostridia (180 OTUs). Family Lachnospiraceae was assigned the largest number of OTUs at 46, followed by Ruminococcaceae (28 OTUs) under class Clostridia, and Streptococcaece comprising 2 OTUs under class Bacilli.

Bacteroidetes, the second most abundant phylum, contained 58 OTUs, all classified under Bacteria. Under the class Bacteroidia, the most predominant family, Prevotellaceae, contained 52 OTUs, and Rikenellaceae contained 24 OTUs. The third most abundant phylum included Probacteria (eight OTUs), all classified under class Gammaproteobacteria. Class Gammaproteobacteria was assigned to the family Enterobacteriaceae and Succinivibrionaceae, and these two families accounted for four OTUs in each. The remaining phyla included Fibrobacter (three OTUs) and Fusobacteria (one OTU).

The 17 unique OTUs of buffalo belonged to three phyla: Bacteroidetes (4 OTUs), Firmicutes (12 OTUs) including Bacilli and Clostridia classes, and Fibrobacteres (1 OTU). In cattle, 774 unique OTUs belonged to six phyla: Firmicutes (422 OTUs under four classes: Bacilli, Clostridia, Erysipelotrichi, and Mollicutes). Bacteroidetes (234 OTUs including Prevotellaceae and Rickenellaceae families), Fibrobacteres (99 OTUs), Actinobacteria (7 OTUs including Coriobacterineae and Actinomycineae families), Cyanobacteria (5 OTUs,) and SRI (7 OTUs).

**Figure 3:**
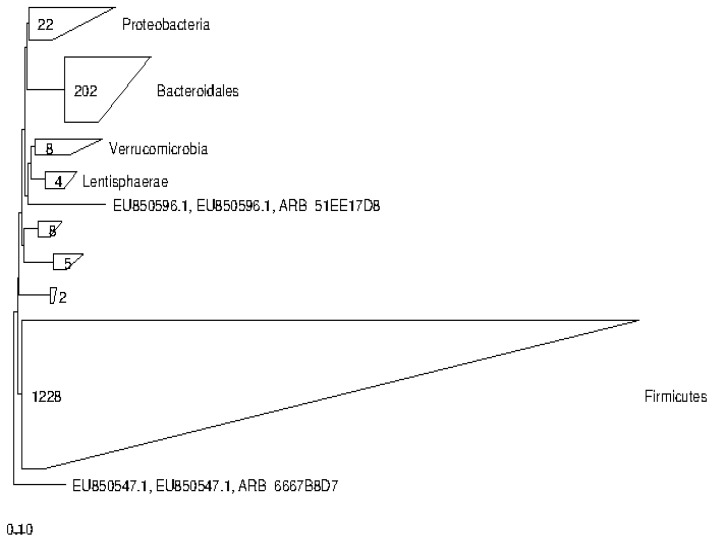
Bacterial phyla represented by the 16S rRNA gene sequences of buffalo rumen origin. The taxonomic tree was created using the ARB program.

**Figure 4:**
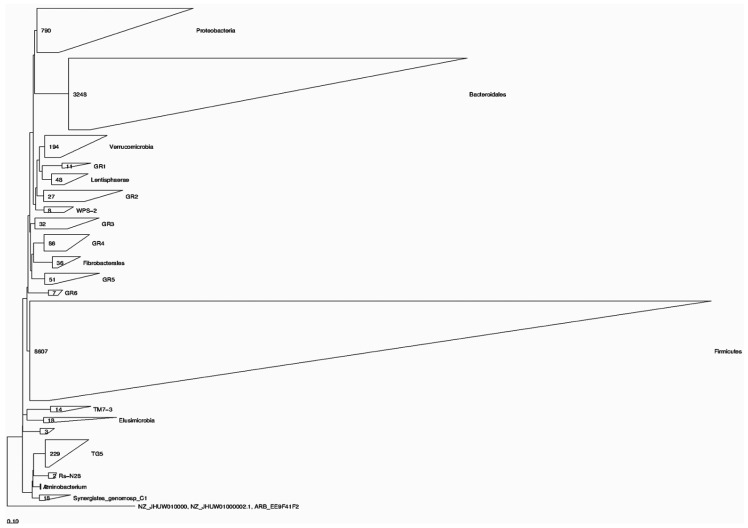
Bacterial phyla represented by the 16S rRNA gene sequences of cattle rumen origin. The taxonomic tree was created using the ARB program.

The phylogenetic placement of sequences retrieved from cattle and buffalo input into the Green genes database, reference ARB tree, using the positional variance by parsimony method is presented in Figure 3 and Figure 4.

In case of buffalo, a total of 1228, 202, and 22 sequences were phylogenetically placed within phyla Firmicutes, Bacteroides, and Proteobacteria, respectively, representing the predominant taxonomic groups.

In case of cattle, a total of 8607, 3248, 790, 229, and 194 sequences were phylogenetically placed within phylum Firmicutes, order Bacteroidales, phylum Proteobacteria, an unclassified taxonomic group TG5, and phylum Verrucomicrobia, representing the predominant taxonomic groups.

## 4. Discussion

In this study, sequences were from diverse studies, and, hence, many sequences were nonoverlapping, which made OTU binning a challenging task. In order to circumvent the problem, we utilized the TreeChopper algorithm (available at https://sourceforge.net/projects/microbiomeutil/files/TreeChopper), a tool developed at the Broad Institute, USA, specifically for OTU binning of nonoverlapping sequences. Using this approach, full-length reference sequences are added with query sequences to facilitate the overlap required for alignment and phylogenetic placement of sequences. Subsequently, it uses a combination of phylogenetic distance and Jaccard similarity to group similar query sequences into OTUs, providing a way to bin sequences even when they are not overlapping.

Similarly, we utilized the ARB software package version 6.0.6 developed by the Technical University of Munich and maintained by Max Planck Institute of Marine Microbiology, Bremen, Germany, to generate a phylogenetic tree using the positional variance by parsimony method for our sequences. It has a central database of processed sequences and allows alignment and placement of query sequences (even if query sequences are nonoverlapping among themselves) in a phylogenetic tree and export of the desired part of the placed sequences as a phylogenetic tree. Similarly, we utilized a sequence-composition-based taxonomic classification tool, RDP classifier, for taxonomic classification of sequences, which does not require overlapped sequences.

A few studies have been conducted to compare rumen bacterial diversity in cattle and buffalo fed the same feeds under controlled experiments, where each group had four to six individuals [27,28], but results of such studies tended to lack convergence. For example, Iqbal et al. [28] reported that the abundance of Firmicutes was higher while the abundance of Bacteroidetes was lower in buffalo, whereas the abundances of the genus *Prevotella* were higher in Jersey cows [37]. Malik et al. [28] reported a total of 43 phyla, 200 orders, 458 families, and 1722 microbial genera in a study involving MiSeq of the rumen metagenome of cattle and buffalo fed the same feed. In their study, *Bacteroidetes* was the most prominent bacterial phylum and constituted >1/3 of the ruminal microbiota; however, their abundances were comparable between cattle and buffaloes. Firmicutes were the second most abundant bacteria, found to be negatively correlated with Bacteroidetes. They inferred that the microbiota community structure and methane emissions were under the direct influence of the feeds and environment, and the host species played only a minor role. This does not agree with our findings. Generally, community compositions uncovered in different individual studies vary due to differences in animal genotype, feed, sample harvesting method, primer sets used, sequencing platform used, and bioinformatic analysis.

Some metagenomic studies focused on the microorganisms present in rumen fluid [38], while other studies analyzed microorganisms associated with solid digesta or both fractions [39]. Such differences may lead to recovery of different sets of microbes from the same animal. NGS platforms provide a large amount of data but the error rate (about 0.1–0.15%) is higher than that of the traditional Sanger sequencing platform (error rate of 0.001%). Although high coverage assembly can reduce sequencing errors for abundant sequences, low-abundance sequences are often discarded as sequencing error [40].

As mentioned earlier, the dataset used in this study included sequences recovered from different geographies and animals fed different feeds, and hence this analysis enabled a fresh and broad view on the global diversity of rumen bacteria of cattle and buffaloes. Our study identified many unique OTUs in cattle and buffaloes, suggesting that the host plays a very important role in defining the rumen microbial community. This is in agreement with an earlier study involving gut fungi, where it was shown that the effect of the host was higher than that of the feed [41]. However, our study had certain limitations. Because of the nature of the data, beta diversity analysis, differential abundance analysis, or statistical comparison of alpha diversity indices could not be performed, as such analyses require replicates.

Further, it should be noted that the current coverage estimated with the present data might be an underestimate, especially in case of buffalo, because, with an increasing number of sequences, the predicted maximum richness tended to increase [42]. The uneven sample sizes may bias comparisons, and the lower rarefaction coverage in buffaloes (78.9%) suggests incomplete microbial representation. Additionally, the lack of dietary or environmental metadata limits the interpretation of observed microbial differences. Future research should include balanced sampling, standardized sequencing protocols, and detailed host metadata (e.g., feed, age, health status) to improve reliability. Functional analyses (e.g., metagenomics or metabolomics) could further clarify the role of microbial differences in digestion efficiency between species.

Our analysis of the sequences of both cattle and buffalo origin identified the classes Firmicutes, Bacteroides, and Proteobacteria as predominant phyla. The other phyla included Fibrobacter, Verrucomicobia, Lentisphaerae, and Sphirochates, which can be considered as minor phyla. Genus *Butyrivibrio*, *Prevotella*, *Psychrobacter*, and *Fibrobacter* were predominant in cattle sequences. In contrast, buffalo rumen was dominated by *Succiniclasticum*, *Prevotella*, *Rumenobacter*, and *Fibrobacter* [43,44]. The *Butyrivibrio* group, which is known to be associated with the biohydrogenation of unsaturated fatty acids, was found to be present in the rumen in large numbers in cattle sequences. Within the class Clostridia, the *Pseudobutyrovibrio* and *Ruminococcus* genera were also prominent in both species. *Ruminococci* are considered to be strongly cellulolytic and can be manipulated genetically or ecologically for increased ruminal cellulolysis. *Succiniclasticum* was the largest genus under the phylum Firmicutes of buffalo sequences. *Succiniclasticum ruminis*, a common rumen bacterium in pasture-fed cows, converts succinate to propionate as its sole energy-yielding mechanism [45], which is considered as a growth-promoting metabolite. Both cattle and buffalo sequences shared a major genus, *Prevotella*, within the class Bacteroides. Another important genus, *Fibrobacter*, considered the main fibrolytic bacterium, was found to be prevalent in good numbers in both cattle and buffalo.

Noronha et al. [46] identified 61 genera from the content of the rumen of buffaloes reared in the Amazon. *Prevotella*, *Succiniclasticum*, *Bacteroides*, *Butyrivibrio*, and *Ruminococcus* were the most prevalent genera of microorganisms. The buffalo rumen microbiome exhibited minor variations across ecosystems and seasons. In another study, dairy buffaloes exhibited significantly different bacterial species, enriched KEGG pathways, and CAZymes encoded genes compared to dairy cows [47]. Holstein cattle and water buffaloes showed distinct ruminal microbiota and nutrient metabolism during suckling, despite comparable growth performance. The rumen of water buffalo was reported to be rich in Firmicutes and fibrolytic bacteria, which accounted for the increased intake of grass and acetate production [48].

Although a large number of bacterial species at the OTU level were identified in the current study, more sequencing efforts need to be undertaken to improve coverage of ruminal global diversity in the case of buffalo. The cattle dataset indicated that the current sequencing effort achieved a satisfactory level of coverage of the diversity of rumen bacteria.

## 5. Conclusions

This study generated substantial information on the diversity of bacteria in the rumen of buffaloes and cattle and demonstrated large variations in the diversity of rumen bacteria between the species, which could enable the development of species-specific strategies for the efficient utilization of fibrous feeds. The genera *Butyrivibrio*, *Prevotella*, *Psychrobacter*, and *Fibrobacter* were documented as most predominant in cattle; however, *Succiniclasticum*, *Prevotella*, *Rumenobacter*, and *Fibrobacter* were the most predominant in buffalo. Further studies should examine how dietary and environmental factors interact to shape rumen microbiota in buffaloes.

## Figures and Tables

**Table 1:** Diversity indices of archaeal 16S rRNA gene sequences retrieved from the rumen of cattle and buffalo.

Attributes	Species-Level Distance (0.03)	
	Buffalo	Cattle
Total number of sequences	1481	13,432
Indices based on all OTUs		
Number of observed phylotypes (OTUs)	1133	5616
ACE	16,188	31,881
Chao1	6008	16,867
Shannon index	6.74	7.78
Simpson index	0.001952	0.001462
Rarefaction richness	3962	9831
% Coverage based on rarefaction	28.6	57.1
Indices based on abundant OTUs (OTUs with at least 3 members)		
Total number of sequences	660	9273
Number of observed phylotypes (OTUs)	189	946
ACE	408	949
Chao1	313	949
Shannon index	4.77	6.28
Simpson index	0.013979	0.003513
Rarefaction richness	239	949
% coverage based on rarefaction	78.9	99.6

OTUs = operational taxonomic units; ACE = abundance-based coverage estimator; Chao1 = an indicator of species richness.

## Data Availability

The data supporting this study’s findings are available from the first author and the corresponding authors upon reasonable request.
